# Correlated Electrical Conductivities to Chemical Configurations of Nitrogenated Nanocrystalline Diamond Films

**DOI:** 10.3390/nano12050854

**Published:** 2022-03-03

**Authors:** Abdelrahman Zkria, Hiroki Gima, Eslam Abubakr, Ashraf Mahmoud, Ariful Haque, Tsuyoshi Yoshitake

**Affiliations:** 1 Department of Applied Science for Electronics and Materials, Kyushu University, Fukuoka 816-8580, Japan; hiroki_gima@kyudai.jp (H.G.); abubakr_eslam@kyudai.jp (E.A.); 2 Department of Physics, Faculty of Science, Aswan University, Aswan 81528, Egypt; 3 Department of Electrical Engineering, Faculty of Engineering, Aswan University, Aswan 81542, Egypt; 4 Advanced Functional Materials and Optoelectronics Laboratory (AFMOL), Department of Physics, College of Science, King Khalid University, Abha 61413, Saudi Arabia; dr_ashraf9@yahoo.com; 5 Ingram School of Engineering, Texas State University, San Marcos, TX 78666, USA

**Keywords:** diamond films, nanodiamond composite, physical vapor deposition, NEXAFS spectra

## Abstract

Diamond is one of the fascinating films appropriate for optoelectronic applications due to its wide bandgap (5.45 eV), high thermal conductivity (3320 W m^−1^·K^−1^), and strong chemical stability. In this report, we synthesized a type of diamond film called nanocrystalline diamond (NCD) by employing a physical vapor deposition method. The synthesis process was performed in different ratios of nitrogen and hydrogen mixed gas atmospheres to form nitrogen-doped (*n*-type) NCD films. A high-resolution scanning electron microscope confirmed the nature of the deposited films to contain diamond nanograins embedded into the amorphous carbon matrix. Sensitive spectroscopic investigations, including X-ray photoemission (XPS) and near-edge X-ray absorption fine structure (NEXAFS), were performed using a synchrotron beam. XPS spectra indicated that the nitrogen content in the film increased with the inflow ratio of nitrogen and hydrogen gas (*I_N/H_*). NEXAFS spectra revealed that the σ*C–C peak weakened, accompanied by a π*C=N peak strengthened with nitrogen doping. This structural modification after nitrogen doping was found to generate unpaired electrons with the formation of C–N and C=N bonding in grain boundaries (GBs). The measured electrical conductivity increased with nitrogen content, which confirms the suggestion of structural investigations that nitrogen-doping generated free electrons at the GBs of the NCD films.

## 1. Introduction

Diamond material has superior properties, including a wide bandgap, the highest hardness, high thermal conductivity, biocompatibility, and chemical stability, as well as several distinctive physical and chemical properties [1,2]. Recently, diamond has triggered more attention for new optoelectronics and high-temperature, high-frequency devices [3,4]. In various applications, elemental nitrogen and phosphorus are the candidate dopants for different materials, including carbon nanotubes [5], graphene [6], transition metal dichalcogenides [7,8], and carbon quantum dots [9]. However, in a single-crystalline diamond, the strongly bonded carbon atoms (with *σ*-bonds) cause a challenge of formation of the *n*-type diamond by nitrogen or phosphorus doping at room temperature due to their high activation energies [10,11]. On the other hand, diamond-like carbon (DLC) film is considered a high potential candidate for various technological applications in electronics and photonics [12]. However, DLC film’s large defect density and complex structure deteriorate the doping processes and control the conduction types.

In contrast, nanocrystalline diamond (NCD) films have attracted substantial interest as a potential material for device-oriented applications [13,14]. NCD films are composed of diamond nanograins which are embedded in an a-C:H matrix. This unique structural configuration possesses properties between diamond and DLC films, and it enables the realization of low activation energy *n*-type doping with nitrogen.

Extensive theoretical and experimental research has been carried out in nitrogen-doped NCD films to unveil the doping mechanism and explore their various applications. In an early study, the theoretical tight-binding molecular dynamics simulations suggested that nitrogen atoms incorporated into NCD films are mainly bonded to the grain boundaries (a-C) rather than the diamond grains [15]. The experimental results of CVD-grown NCD films, as reported by Ikeda et al. [16], disclosed the increment of sp2 at the expense of sp3 by nitrogen doping. They stated that nitrogen doping would be associated with forming some midband states, particularly: *π* and π* states for sp2-bonded atoms, and *σ* and σ* states for sp3-bonded atoms. In agreement with the suggestion of Ikeda et al., our previous report [17] revealed the shrinkage of the optical band gap of NCD films after nitrogen incorporation due to the formation of midband states.

Regarding the electrical properties, Birrell et al. suggested that nitrogen might create additional electric conduction paths in NCD films, resulting in an enhanced electrical conductivity due to increased sp2 bonds [18]. In our earlier research works, the *p*-*n* heterojunction diodes were fabricated by integrating the commercially supplied (*n*- or *p*-type) Si substrates either to *p*-type (B-doped) or *n*-type (N-doped) NCD films, respectively [19,20]. The 3 at.% N-doped NCD films deposited on *p*-type Si were experimentally investigated by current–voltage characteristics, and the results revealed a rectification ratio with four orders of magnitude [20]. Furthermore, we investigated the origin of dark current in the fabricated heterojunction based on an established comprehensive computer device model [21]. Most recently, we employed the impedance spectroscopy analysis to provide a prominent separated contribution of sp3 and sp2 bonded carbons to the capacitance values of 3 at.% N-doped NCD films [22].

In prior reports, we investigated the N-doped NCD with fixed nitrogen concentrations (3 at.%). Therefore, the objective of the present research work is to elucidate the influence of changing the nitrogen concentration on the electrical conductivities of NCD films and correlating them with the chemical structure configurations. In this regard, the NCD films were synthesized by a physical vapor method, called coaxial arc plasma gun. The influence of nitrogen doping with different values was electrically characterized by the four-probe method. Furthermore, the synthesized films were intensively examined by sensitive spectroscopic measurement tools including X-ray photoemission and near-edge X-ray absorption fine-structure spectroscopies measured with synchrotron radiation available at Kyushu University Synchrotron Center in Saga, Japan.

## 2. Experimental Details

### 2.1. Synthesize Nanodiamond Composite Film by Physical Vapor Deposition

**Figure 1:**
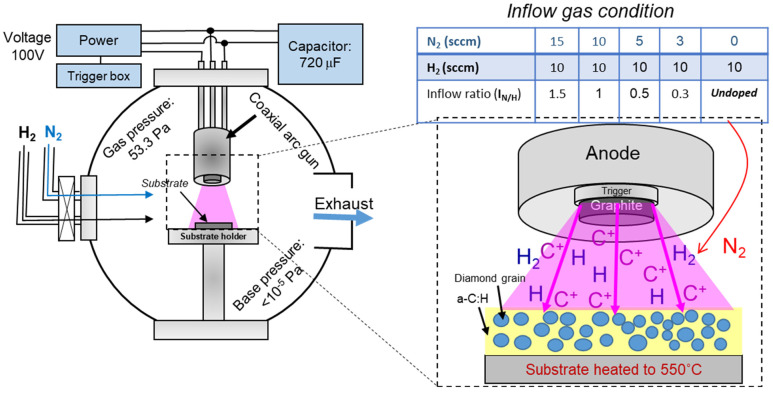
Schematic illustration of coaxial arc plasma gun technique. Upper table shows the inflow ratio of nitrogen/hydrogen gases to form nitrogen-doped films.

Nanodiamond composite (NCD) film was grown on commercial *p*-type mirror-polished Si (100) (SUMCO corporation) substrates by coaxial arc plasma gun (CAPG) approach. In this fabrication method, a CAPG (Ulvac ARL-300, Ulvac, Inc., Chigasaki, Japan) is equipped with a bulk graphite target (purity of 99.9%, Toshima Manufacturing Co., Ltd., Higashimatsuyama, Japan). Prior to the film growth, the surface of Si substrates was cleaned by acetone and methanol solvents followed by deionized (DI) water for 7 min, then dried by N_2_ gas and finally fixed on the top of the substrate holder, facing the graphite target at a distance of 15 mm. Inside the chamber was evacuated up to 10^−6^ Pa via a turbomolecular pump (TG450FVAB, Osaka Vacuum Co., Ltd., Osaka, Japan) equipped with a rotary pump (Edwards E2M80, Edwards Vacuum, Yachiyo, Japan). The substrate’s temperature was elevated up to 550 °C. As shown in Figure 1, mixed gases of H_2_ and N_2_ with a purity of 3N (Sumitomo Seika Chemicals Co., Ltd., Osaka, Japan) were slowly fed into the chamber through apertures, and the final deposition pressure was kept at 53 Pa. During the film growth, the applied voltage to the coaxial arc plasma gun and the discharge pulse repetition rate were maintained at 100 V and 5 Hz, respectively.

### 2.2. Characterizations of the Synthesized Nanodiamond Composite Films

An advanced high-resolution scanning electron microscope (HRSEM) was employed for the phase identification of synthesized nanodiamond composite films. For this purpose, a JSM-IT7700HR (JEOL Ltd., Tokyo, Japan) was operated with an acceleration voltage of 15 kV and 10,000× magnification to photograph the top view of the N-doped NCD films grown on Si substrate. In addition, the surface roughness of N-doped NDC film was observed at room temperature by atomic force microscopy (AFM, Nano-RTM, close contact mode). Furthermore, X-ray photoemission spectroscopy (XPS) was used to track the synthesized films’ nitrogen amount, which varied with the nitrogen/hydrogen inflow ratio (*I_N/H_*) during deposition. In the current study, *I_N/H_* was changed from 0.3 to 1.5, and XPS measurements were carried out using the MgKα line (source energy of 1253.6 eV). Additional chemical bonding investigations were examined by near-edge X-ray absorption fine-structure spectroscopy (NEXAFS) using synchrotron radiation with an incident photon energy of 350 eV at beamline 12 of the Kyushu Synchrotron Light Research Center (SAGA LS). Synchrotron radiation possesses various remarkable properties, which make it an attractive technique for quantitative measurements. The electrical conductivities were measured by the van der Pauw method. In this method, the sample was prepared in well-defined squared geometries. The resistivity is calculated according to the following equation:

(1) $$\rho =\frac{\pi \cdot t}{2\cdot \ln\left(2\right)}\cdot \left[\frac{V_{43}+V_{23}}{I}\right]\cdot F\cdot Q$$

where *I* is the constant current applied, *t* represents the active layer thickness, *F* and *Q* are the correction factors for geometrical asymmetry and symmetry factors, respectively. *F* and *Q* were calculated as follows:

(2) $$Q=\frac{V_{43}}{V_{23}}\;,\;F=1-0.3465A-0.09236A^{2},\;A={\left[\frac{Q-1}{Q+1}\right]}^{2}$$

Accordingly, the activation energy was calculated to be shown on results and discussion.

## 3. Results and Discussion

### 3.1. Morphology and Nitrogen Contents of N-Doped Nanodiamond Composite Films: By SEM, AFM, and XPS

**Figure 2:**
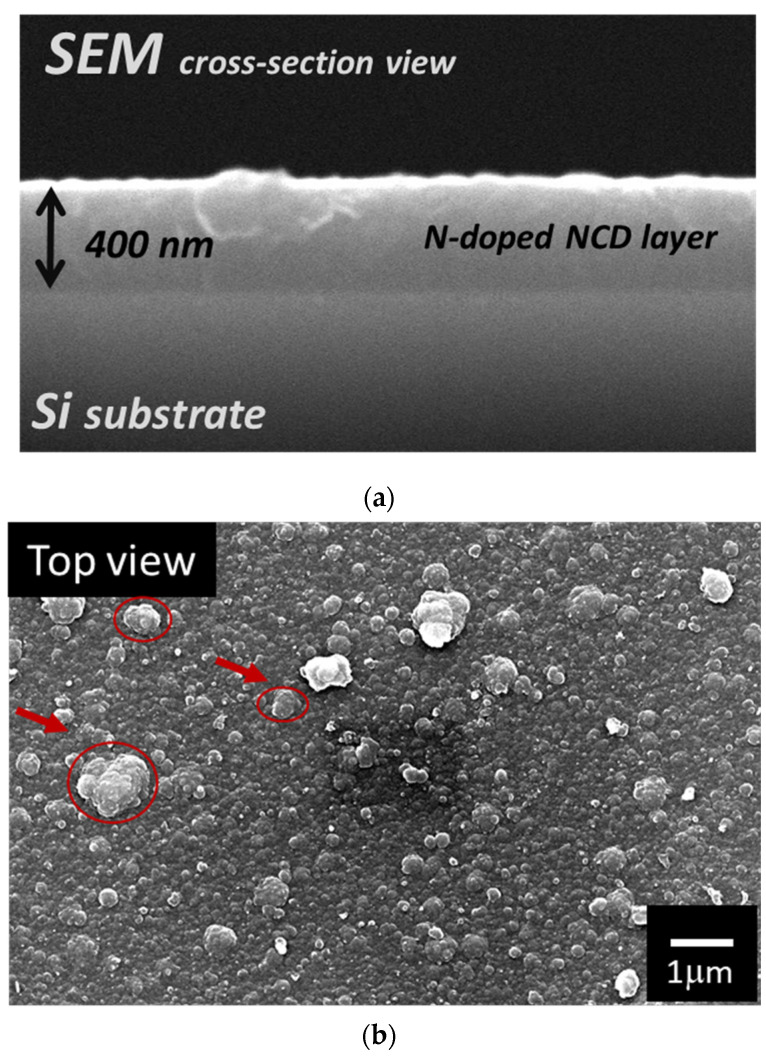

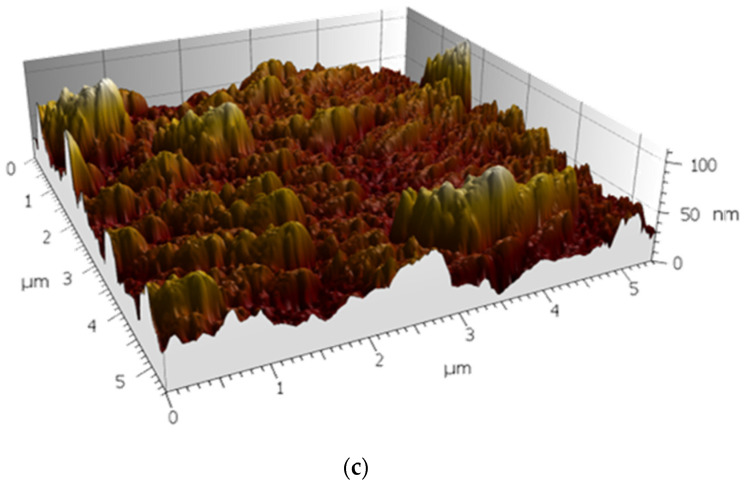
(**a**) Cross-sectional; (**b**) top-view SEM images of nitrogenated nanodiamond films; (**c**) topographic 3D-AFM image for the smooth surface of nitrogenated nanodiamond films.

SEM and AFM images explored the morphology and topology of the synthesized nanodiamond composite films. The results revealed uniform and homogeneous films coated onto the top of Si substrates. In Figure 2a, the SEM image illustrates the film morphology composed of diamond nanograins with different sizes (marked in red circles); those diamond nanograins are embedded into the amorphous carbon (a-C) matrix. The film was smoothly deposited on the top of mirror-polished Si substrates without any cracks or bindings at the film/Si interface, as clearly shown in Figure 2b. A typical AFM image of the NCD film, presented in Figure 2c, indicates a surface with root mean square (RMS) roughness of 8 nm. The roughness of the observed surface of the grown NCD film was much smaller than the reported results by Sharda et al. [23] of nanodiamond films deposited by chemical vapor deposition, which had a roughness of 16 nm.

**Figure 3:**
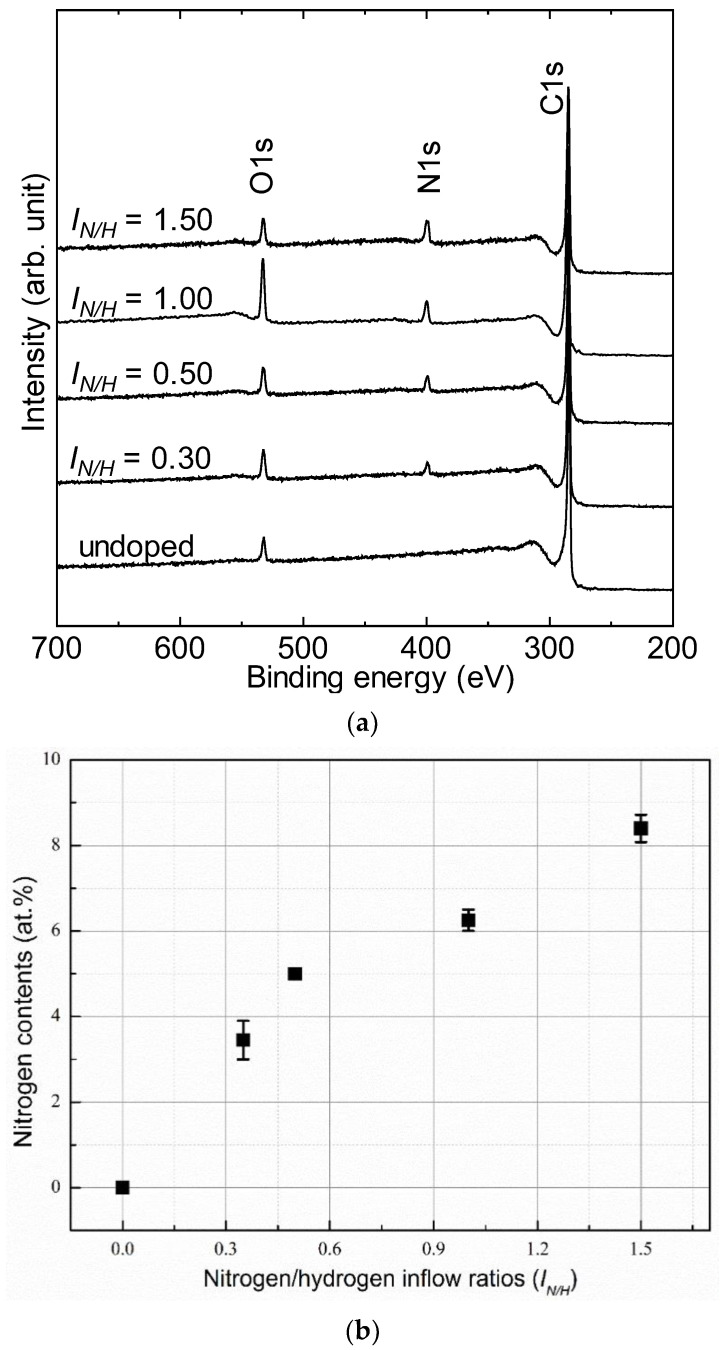
(**a**) X-ray photo emission spectrum of nitrogenated nanodiamond films deposited at inflow ratio of *I_N/H_* = 0, 0.3, 0.5, 1.0, and 1.5; (**b**) calculated nitrogen contents in the synthesized films.

X-ray photoemission spectroscopy is a powerful technique that can precisely explore the chemical structure of thin films surfaces by detecting photo-generated electrons from the C1s core level. Figure 3a shows the wide-scan XPS spectra of the nanodiamond composite films prepared at different nitrogen/hydrogen inflow ratios. All films exhibited a sharp intense peak located at a binding energy of 284 eV, which originated from the collected photoelectrons of excited core levels of carbon atoms and is known as the C1s peak. Additionally, a non-negligible peak was observed at a binding energy of 532 eV; this is mainly attributed to the adsorbed oxygen atoms at surface-prepared thin films due to the exposure of samples to the laboratory atmosphere, known O1s peak [24]. Unlike the deposited film in only the hydrogen gas atmosphere, all other films revealed an extra peak centered at a binding energy of 409 eV, which is assigned to excited core levels of nitrogen atoms (N1s) [25]. The detected N1s peak declares the contribution of nitrogen atoms from the hydrogen/nitrogen mixed gas atmosphere into the synthesized NCD film. Moreover, the intensity of N1s peak was increased by increasing the nitrogen gas flow rate during the deposition. Quantitatively, the amount of nitrogen in the film can be estimated using the following equation:

(3) NC=NareaNpNareaNp+CareaCp

where *N_area_* and *C_area_* are the total areas of the N1s and C1s peaks, and *N_p_* and *C_p_* are the photoionization cross-sections of N1s and C1s, respectively. The values of *N_p_* and *C_p_* are 1.8 and 1.00, respectively [26]. The relationship between the nitrogen contents and flow ratio resulting from the calculation is presented in Figure 3b.

### 3.2. Electrical and Chemical Bonding Analysis of N-Doped Nanodiamond Composite Films

**Figure 4:**
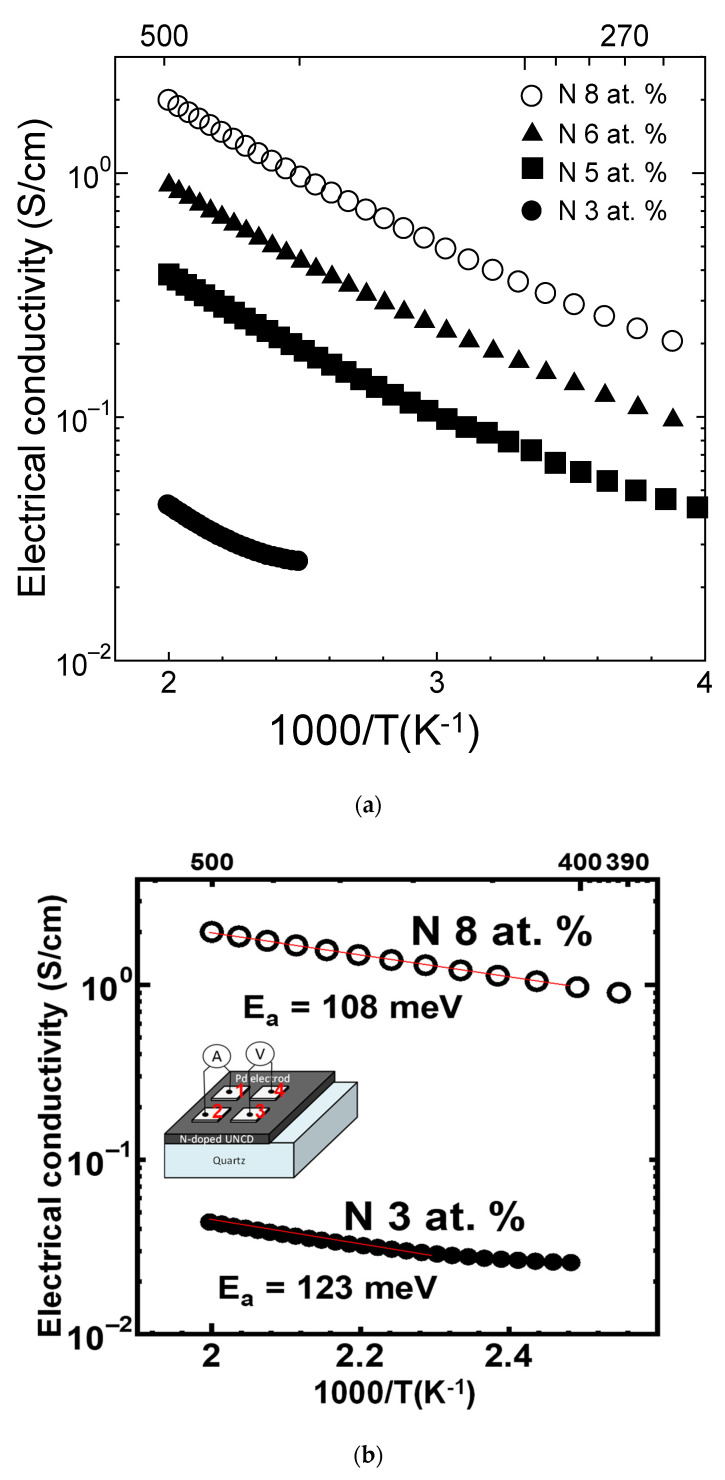
(**a**) Arrhenius plot of electrical conductivity of different nitrogenated nanodiamond films; (**b**) the fitting of minimum and maximum values of nitrogen contents (3 at.% and 8 at.%) to calculate the activation energies.

To examine the influence of nitrogen doping with different concentrations on the electrical properties of the deposited NDC films, the temperature dependence electrical conductivities were measured. The electrical conductivities (*σ*) of all prepared films, deposited on a quartz substrate, were evaluated by the van der Pauw method. Figure 4a illustrates the increased electrical conductivity with increasing nitrogen contents into the films. In addition, the electrical conductivities increased with elevated temperature, which affirms the semiconducting behavior of the synthesized N-doped NCD films. Figure 4b depicts the log *σ* vs. 1/*T* plot for minimum and maximum values of nitrogen contents (3 at.% and 8 at.%), expressed by

(4) σ=σ0 exp(−EaKBT)

The activation energy of each film was estimated from the Arrhenius plot to be 123 meV and 108 meV for 3 at.% and 8 at.% nitrogen-doped NCD films, respectively.

**Figure 5:**
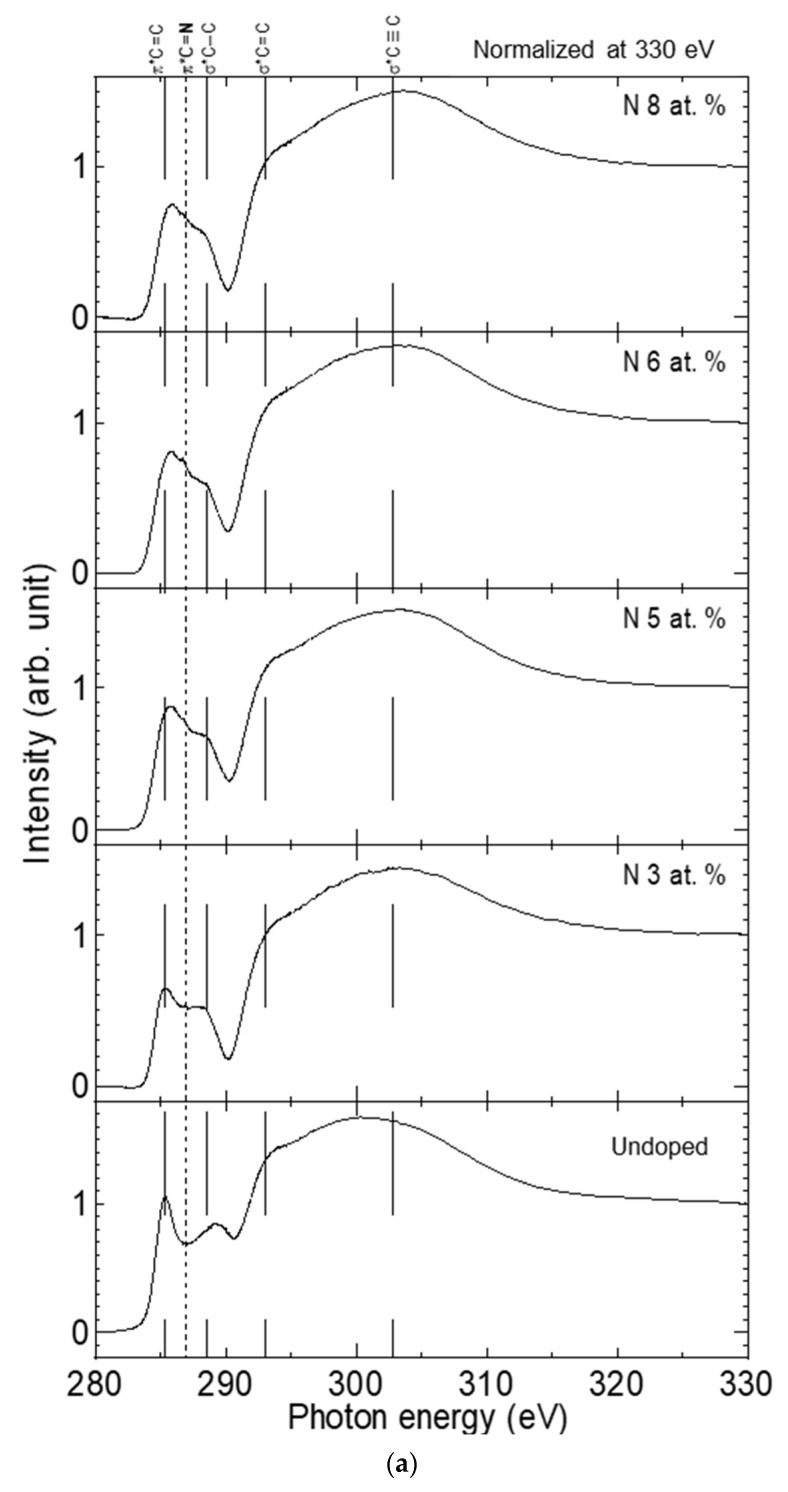

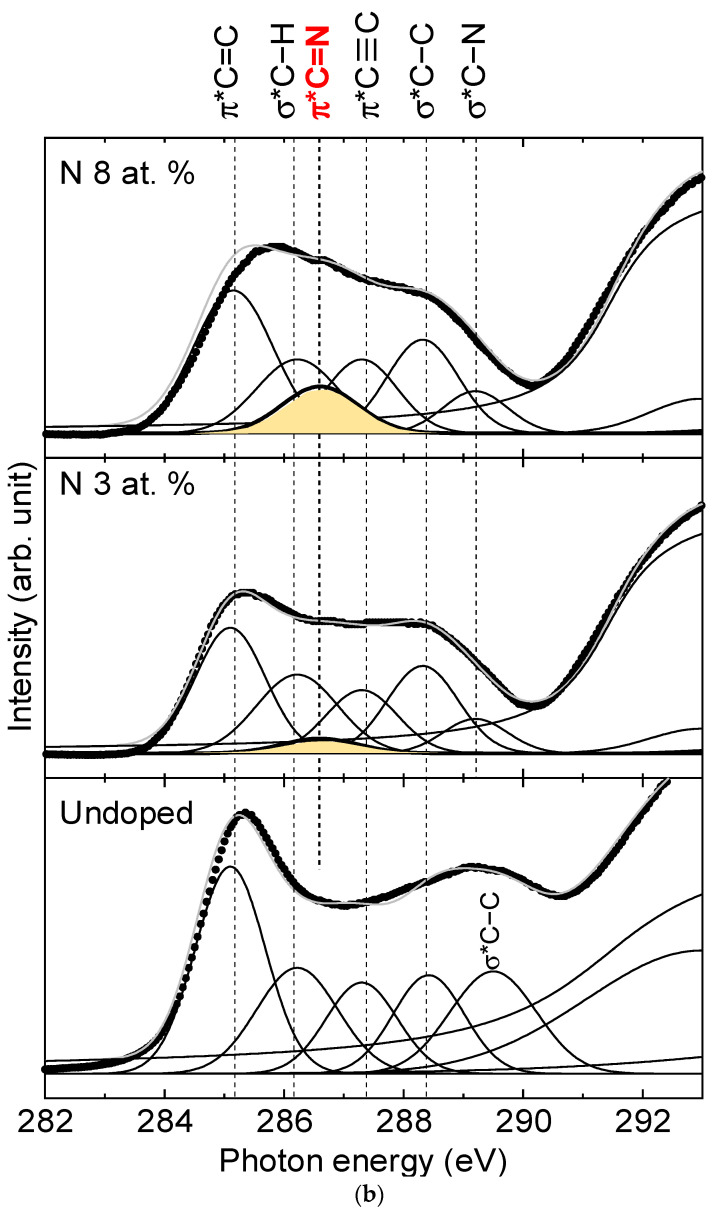
(**a**) C K-edge NEXAFS spectra of nitrogenated nanodiamond films deposited at different inflow ratios; (**b**) the deconvoluted NEXAFS spectra of minimum (3 at.%) and maximum (8 at.%) values of nitrogen contents.

Synchrotron radiation spectroscopy is known as a sensitive tool that can be employed to detect the structural evolution of various carbon nanomaterials. Effectively, NEXAFS can probe the final-state wave function near the excited atom, wherein transitions from the C1s core level to the area of the unoccupied state are caused by X-ray photon absorption. Figure 5 shows the C K-edge NEXAFS spectra for all the nanodiamond composite films grown on Si substrates in the hydrogen/nitrogen atmosphere. Similar to the literature [27], the NEXAFS spectra were normalized in intensity at 330 eV, and all spectra revealed two prominent peaks: one sharp absorption peak located at a photon energy of 285 eV due to π* transition (π*C=C) bond, and another peak located between 290 and 295 eV corresponding to σ* (σ* C=C) bonding structure [28]. Compared to the undoped film (only in H_2_ atmosphere), all nitrogen-doped NCD films (3, 5, 6, 8 at.%) exhibited three additional components, as shown in Figure 5a. For more analysis of peak components, NEXAFS spectra of minimum (3 at.%) and maximum (8 at.%) values of nitrogen contents were deconvoluted into component peaks, with the similar processes described in our previous work [29], as presented in Figure 5b. In Figure 5b, the NEXAFS spectra with peak positions at 286.7 and 289.2 eV originated from π*C=N and σ*C−N [30]. Obviously, the π*C=N peak is strengthened with increasing nitrogen content in the films from 3 at.% to 8 at.%; Lopez et al. [31] assigned the peak originating from π*C=N to the shoulder at 286.6 eV. On the other hand, the strengthening of the π*C=N peak is accompanied by a suppression of σ* C–C peak intensity. This observation indicates the conversion of C–C sp3 bonds to C–C sp2 bonds by increasing nitrogen contents in the NCD films from 3 at.% to 8 at.%. The sp3 hybridized carbon, including the nanodiamond grains in the films, were represented by intense *σ*1* C–C (288.29 eV) and *σ*2* C–C (289.3 eV) peaks with a stronger representation at *σ*1 C–C [32]. In the case of the hydrogenated samples, the peak assignments were not all consistent due to new bonding possibilities. The NCD(:H) film exhibited a larger single σ*C–C peak than the non-hydrogenated film [27], considering the appearance of the σ*(C–H) at 289.3 eV as reported by Kanda et al. [33,34]. With nitrogen incorporation, the peak of σ* C–N appeared and was considered dominant, especially because hydrogen atoms at GBs are replaced by nitrogen atoms in the nitrogen-doped films; this is confirmed by the weakening of the *sp*3-CH upon nitrogen incorporation. However, this peak slightly depends on nitrogen incorporation because the σ*(C–N) is not expected to build a well-defined molecular structure [35]. Therefore, this sharp dominant peak is interpreted to originate from the transition to a π* state. Guo et al. assigned this peak to transitions from the C1s level to unoccupied π* states of the C=N bond [36]. The analysis of sensitive NEXAFS spectra suggests that π*C=N, which originated from nitrogen dopants, is formed at grain boundaries, and in turn, generates free electrons which are responsible for the enhanced electrical conductivities.

## 4. Conclusions

400nm-thin nanodiamond composite films were grown on Si substrates in mixed hydrogen/nitrogen atmospheric gas by an arc plasma gun. X-ray photoemission spectra indicated the increment of nitrogen content in the film by increasing the nitrogen flow rate. The electrical analysis confirmed the formation of *n*-type semiconductors by nitrogen doping, and the electrical conductivity increased with the nitrogen concentrations. Sensitive NEXAFS measurements revealed that the σ*C–C peak weakened and the π*C=N peak strengthened with nitrogen doping in the films. By applying the NEXAFS spectra analysis, nanodiamond films’ structural evolution after nitrogen doping was successfully correlated to the enhancement of electrical conductivity.

## Data Availability

All data used to support the findings of this study are included within the article.
